# Autoinflammatory Recurrent Pericarditis Associated with a New NLRP12 Mutation in a Male Adolescent

**DOI:** 10.3390/life13112131

**Published:** 2023-10-28

**Authors:** Eliza Cinteza, Dan Stefan, Mihaela Adela Iancu, Andreea Ioan, Corina Maria Vasile, Radu Vatasescu, Alexis Cochino

**Affiliations:** 1 Department of Pediatrics, “Carol Davila” University of Medicine and Pharmacy, 020021 Bucharest, Romania; eliza.cinteza@umfcd.ro (E.C.); alexis.cochino@umfcd.ro (A.C.); 2 Department of Pediatric Cardiology, “Marie Curie” Emergency Children’s Hospital, 41451 Bucharest, Romania; dan-valentin.stefan@rez.umfcd.ro; 3 Department of Internal, Family and Occupational Medicine, “Carol Davila” University of Medicine and Pharmacy, 020021 Bucharest, Romania; 4 Department of Pediatrics, “Alessandrescu Rusescu” National Institute for Mother and Child Health, 020395 Bucharest, Romania; berariuandreea@gmail.com; 5 Pediatric and Adult Congenital Cardiology Department, M3C National Reference Centre, Bordeaux University Hospital, 3300 Bordeaux, France; corina.vasile93@gmail.com; 6 Cardio-Thoracic Department, “Carol Davila” University of Medicine and Pharmacy Bucharest, 020021 Bucharest, Romania; 7 Department of Cardiology, Clinic Emergency Hospital Bucharest, 050098 Bucharest, Romania

**Keywords:** autoinflammation, recurrent pericarditis, NSAID non-responsiveness, colchicine resistance, corticoid dependence, NLRP12 mutation

## Abstract

Idiopathic recurrent pericarditis (IRP) can be the hallmark of an autoinflammatory syndrome with recurrent attacks of chest pain and symptom-free intervals following an acute episode. The recurrence rate may be 35% in the pediatric population, frequently with less severe manifestations than at the first episode. Pericarditis can be the sole clinical manifestation or may be part of a systemic autoinflammatory disease (SAID), especially in the case of a recurrence. Familial Mediterranean Fever (FMF), Tumor Necrosis Factor Receptor-Associated Periodic Syndrome (TRAPS), Mevalonate-Kinase Deficiency (MKD), nucleotide-binding oligomerization domain 2 (NOD2)-associated autoinflammatory syndrome, and others are closely related to IRP based on similar clinical manifestations and treatment responses to anti-interleukin 1 (IL-1) agents, such as anakinra, and should therefore be excluded in patients with IRP. A newly described SAID, an autosomal dominant disorder known as NLRP12-AID (nucleotide-binding leucine-rich repeat-containing receptor 12-related autoinflammatory disease) is caused by heterozygous mutations in the NLRP12 gene and most commonly affects children. Fewer than 40 pediatric patients with NLRP12-AID have been described in the medical literature, with none presenting with RP. We report a case of relapsing pericarditis responsive to anti-IL-1 therapy in a male adolescent who carried a missense mutation in the NLRP12 gene potentially causative of the excessive activation of inflammatory pathways. This is a unique case in the medical literature that associates recurrent pericarditis in an adolescent presumed to be related to the missense mutation in the NLRP12 gene. The role of the NLRP12 inflammasome in generating and maintaining recurrent pericardial inflammation should be considered.

## 1. Introduction

Pericardial diseases may occur in isolation or as part of a systemic condition (e.g., autoimmune or autoinflammatory disease). The primary pericardial syndromes encountered in clinical practice include pericarditis (acute, incessant, recurrent, and chronic), cardiac tamponade, constrictive pericarditis, and pericardial masses. Pericarditis is defined as the inflammation of the pericardial layers, being mostly idiopathic, although it may result from infectious and non-infectious causes [1,2,3]. The etiology remains unknown in up to 55% of the cases diagnosed in Western countries [4]. An established diagnosis of RP requires a documented first flare, a symptom-free interval of at least 4–6 weeks, and the presence of recurrent episodes of pericarditis. Pericarditis relapse rate ranges from 15 to 30%, with less severe manifestations than the first episode [5,6] which may increase to 50% after the first recurrence in patients without prophylactic treatment (e.g., with colchicine) [7,8]. Therefore, multiple recurrences of pericardial inflammation that are unresponsive to standard treatment should prompt investigations for a dysregulated immune response and raise the suspicion of a distinct entity such as a systemic autoinflammatory disease (SAID) [9,10,11].

The first disease included in the group of SAIDs was familial Mediterranean fever, which was added in 1997, and since then the number of SAIDs has increased to more than 30 [12,13]. The clinical manifestations of SAIDs include recurrent fever, rash, serositis, ocular or joint inflammation, and neurological impairment with no identifiable trigger. In 2022, the International Union of Immunological Societies (IUIS) expert committee documented 56 genetic defects associated with monogenic autoinflammatory diseases and updated the subgroup disorders according to their pathological mechanisms into type 1 interferonopathies, as defects affecting the inflammasome- and non-inflammasome-related conditions (Table 1) [12].

IRP etiopathogenesis hovers on the overlap between autoimmune and autoinflammatory molecular pathways. However, as recent studies focused more on the innate immune system, the activation of the inflammasome became the pathogenic hallmark of RP, with high proinflammatory cytokine levels (e.g., IL-1ß, IL-18) secondary to pyroptosis and a high clinical resemblance with other SAIDs [14]. In addition to imagistic tests, the investigation protocol of IRP includes serologic testing for the exclusion of an immune disease as well as genetic testing for newly detected possible etiologies. There has been a recent shift in understanding of the pathogenesis of RP that is primarily based on the activation of inflammasomes [15,16,17,18,19]. Innate immunity is triggered by pattern recognition receptors (PRRs) that are expressed in a variety of cell lines and are responsible for producing an inflammatory response. PRRs identify signals derived from microbial constituents (PAMPs, pathogen-associated molecular patterns) or damaged tissue components (DAMPs, damage-associated molecular patterns). Certain members of PRRs were found to form inflammasomes, a group of multimeric protein complexes responsible for activating inflammatory pathways, such as the nucleotide-binding oligomerization domain (NOD); and the leucine-rich repeat (LRR)-containing protein (NLR) family members NLRP1, NLRP3, NLRC4, absent-in-melanoma 2 (AIM2), pyrin [20,21], and the less characterized NLRP2, NLRP6, NLRP7, NLRP12, and IFI16 [22,23].

The newly described NLRP12-AID (nucleotide-binding leucine-rich repeat-containing receptor 12-related autoinflammatory disease) is an autosomal dominant disease resulting from mutations in the NLRP12 gene. The NLRP12 gene is an innate immune sensor encoding the monarch-1 protein, a “NOD-like” receptor that regulates the downstream inflammatory process. There have been case reports and small cohort studies published in the last two decades that link NLRP12 mutations to inflammation-triggered manifestations. The number of cases is extremely small around the world, so obtaining statistically significant numbers and pertinent data is very challenging [13]. Up to our case report, NLRP12-AID has not yet been associated with RP. Some genetic variants are clearly associated with the disease, while others are not. The last category includes the variants of uncertain significance (VUS), which may be revealed as pathogenic variants with time.

## 2. Case Report

A 14-year-old male was transferred to our clinic for further investigation after being diagnosed with pericardial effusion at a provincial hospital. Fever and left shoulder pain were the first symptoms of the illness. An examination of the patient revealed a 45 kg male with a normal exam other than a reduction in the intensity of the cardiac sounds. The patient’s heart rate was also found to be elevated. Other clinical manifestations, including arthralgia and abdominal pain, were thoroughly evaluated, and it is noteworthy that these symptoms were conspicuously absent throughout the patient’s clinical course. Despite their absence, the diligent assessment of negative symptoms is paramount in comprehensive patient care.

**Figure 1:**
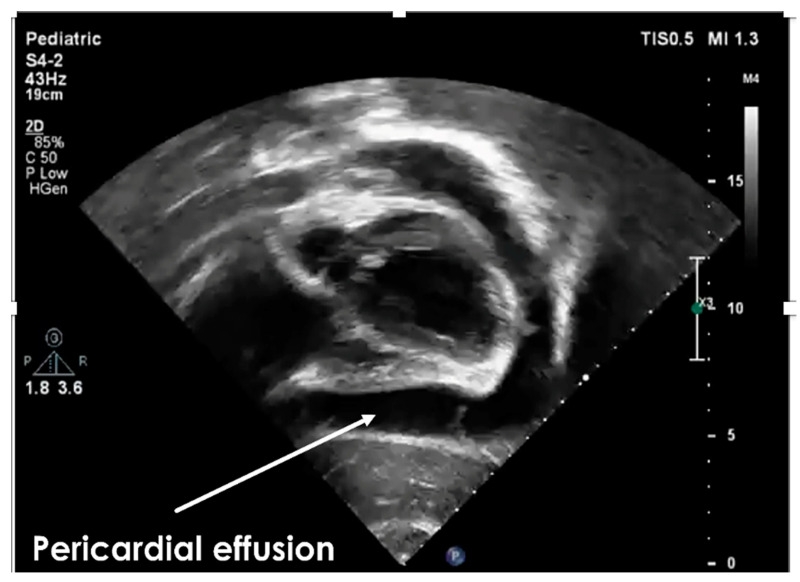
The echocardiographic subcostal view during the first presentation of our subject for acute pericarditis showed moderate pericardial effusion. Legend: white arrow showing pericardial effusion.

Three weeks prior to this event, he had a viral infection of the upper respiratory tract treated with ibuprofen. Echocardiography revealed a moderate pericardial effusion (Figure 1) and a mild bilateral pleural effusion.

Biological investigations showed the white blood cell count was 19,670/µL, the neutrophils count was 16.8/µL, the lymphocytes count was 810/µL, and there were high levels of inflammatory markers in the plasma tests: the C-reactive protein (CRP) level was 131 mg/L, the erythrocytes sedimentation rate (ESR) was 72 mm/h, the rheumatoid factor was positive (20UI/mL, normal value, NV < 14 UI/mL), and the ferritin level was high at 380.3 ng/mL (NV = 14–152 ng/mL). The complement fractions (C3, C4) were normal; autoantibodies were not detected; and the Quantiferon TB Gold, tuberculin skin test, and antibody work-up for common viral infections were all negative. An immunogram showed hypoIgG (652 mg/dL, NV 720–1700 mg/dL). The initial approach was to start with non-steroid therapy. Naproxen 20 mg/day was initiated, but with no response. As the pericardial effusion increased and the patient was symptomatic, corticoid therapy was started at a dose of 60 mg of Prednisone for ten days, tapering down by 5 mg every two weeks.

After twelve weeks, the first relapse occurred when the dose of 20 mg of Prednisone was decreased. The patient complained of chest pain and dyspnea with orthopnea. Tachycardia with low-intensity heart sounds and diminished pulmonary sounds on the left half of the thorax were noticed during clinical examination. Echocardiography revealed a circumferential pericardial effusion of 9–15 mm. Furthermore, a left pleural effusion was associated. The ECG showed PR segment depression and ST elevation in the inferior leads. In addition to a negative antinuclear antibodies (ANAs) profile, a Sanger sequencing for the MEFV gene was performed without any mutations identified. The therapeutic strategy was similar: increasing the Prednisone dose to 30 mg daily.

**Figure 2:**
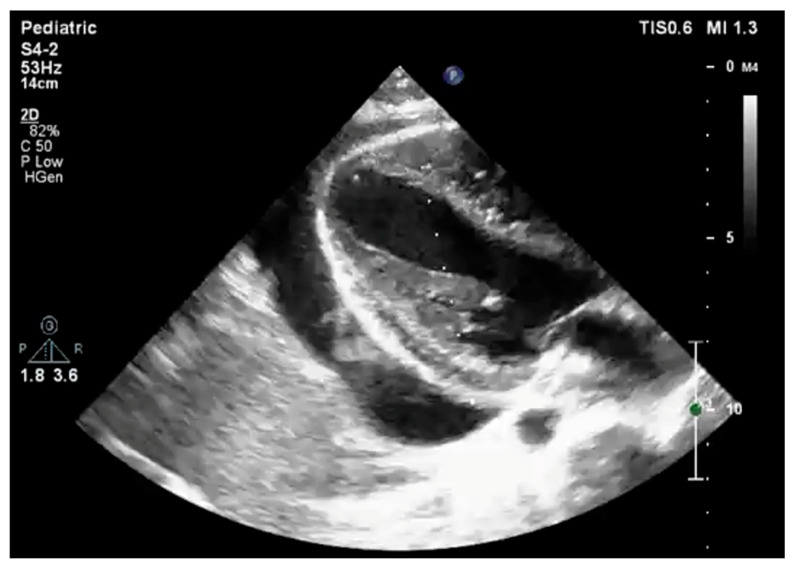
Echocardiographic parasternal long-axis view during the fourth relapse of pericarditis showing severe pericardial effusion.

Approximately six weeks after the first relapse, a second occurred. The corticosteroid therapy associated with colchicine, 1 mg/day, resolved the symptoms and the pericardial effusion. Three months later, at the point of reducing the Prednisone dose below 20 mg per day while he was on Colchicine, the patient severely relapsed for the third time, the effusion measuring 22–25 mm in thickness (Figure 2). An exudative profile was evident in the pericardial liquid extracted through pericardiocentesis. There was evidence of an iatrogenic Cushing syndrome at this point. Since the symptoms only responded to corticosteroids, relapses occurred when the daily dose of Prednisone was decreased below 20 mg per day. The patient began to display signs of chronic corticoid adverse reaction to treatment. An increase in weight gain to 26 kg, an appearance of “round face”, acne, infiltrated skin, and a depressive mood all contributed to poor treatment compliance. In order to wean him off steroids, we added Azathioprine up to the maximum dose of 2.5 mg/kg/day.

Throughout the follow-up period, systemic rheumatic diseases were excluded repeatedly. Azathioprine and colchicine were ineffective in preventing further relapses. A recurrence of pericarditis associated with fever, high inflammatory markers, and a positive but transitory response to corticoid therapy led to the suspicion of SAID. Biological investigations showed an elevated interleukin-1 beta (IL-1β) level in the patient’s blood, with a 10 pg/mL recorded value (normal values < 5 pg/mL). This elevation in IL-1β levels was a significant indicator of the patient’s inflammatory status, supporting the suspicion of autoinflammatory involvement in the pathogenesis of recurrent pericarditis.

The patient started treatment with anakinra, a recombinant interleukin-1 receptor antagonist (anti-IL-1) at 2 mg/kg/day, with remarkable results. He relapsed in the COVID-19 emergency alert period when anakinra was unavailable and the treatment was stopped, but as soon as it was restarted everything went well. With the introduction of anakinra, a remarkable control of disease flares was achieved. Notably, anakinra demonstrated a substantial glucocorticoid-sparing effect in this patient, reducing the need for high doses of prednisone. Before initiating anakinra treatment, the patient was likely to experience disease flares at a prednisone dosage of 20 mg.

Upon introducing anakinra, the patient’s minimum maintenance dose of prednisone while maintaining disease control was significantly reduced, from 20 mg to a dosage of 5 mg, followed by the ending of glucocorticoid therapy while on anakinra.

This glucocorticoid-sparing effect of anakinra not only contributed to the patient’s improved quality of life, but also helped mitigate the potential adverse effects associated with prolonged high-dose corticosteroid therapy, such as iatrogenic Cushing syndrome, weight gain, and other corticosteroid-related adverse reactions.

Next-generation sequencing (NGS) performed by the Invitae Corporation, San Francisco, USA, revealed a previously unreported de novo missense mutation in the NLRP12 gene (c.1264G > A), which was classified as a variant of uncertain significance (VUS). Both parents were genetically tested for the same mutation. Only the father was proved to be positive in the heterozygous form without experiencing symptoms related to pericarditis. A published report [24] provides additional information regarding the patient’s phenotype and the frequency of RP among a cohort of pediatric patients with SAIDS. After a period of two years, the administration of anakinra was reduced to one dose every two days and then to one dose every three days, with no relapses.

## 3. Discussion

According to the first theory regarding the triggers of pericarditis, the adaptive immune system is activated in response to cardiotropic viruses, mycobacteria, or without any obvious trigger [14,25]. As a result of viral or bacterial injury to the heart or distinct environmental factors, self-antigens can be exposed, which then become immunogenic and stimulate CD4+ T lymphocytes to divide into Th1 and Th17 lymphocytes, which in turn release pro-inflammatory cytokines (Il-6, Il-8, IFN alpha). The pericardium is repeatedly damaged, resulting in the production of anti-heart antibodies (AHAs) and anti-intercalated disk antibodies (AIDAs). A few cases were found to have antinuclear antibodies (ANAs), particularly in patients with rheumatic disease. However, the titers of these antibodies were low. Although autoantibodies are often regarded as markers of autoimmunity, they may also be nonspecific consequences of pericardial inflammation. In addition, there is evidence to support the role of the adaptive immune system in treating RP patients with intravenous immunoglobulins or immunosuppressants targeting cell immunity (e.g., azathioprine) [24].

NLRPs (NACHT, LRR, and PYD domains) are proteins expressed primarily in macrophages. They are components of the inflammasome, which serves as a pattern recognition receptor (PRR) for pathogen-associated molecular patterns (PAMPS). Regarding NLRP pathogenesis, the NLRP3 receptor is one of the most studied. Based on the proposed pathogenicity models, pericardial samples from patients with chronic RP who are experiencing symptoms exhibit significantly higher levels of NLRP3 inflammasome expression than those from controls. It has also been demonstrated in murine models that IL-1 blockade significantly improves pericarditis caused by NLRP3 activation. NLRP3 inflammasome plays a key role through its innate protection against viral, bacterial, and fungal infections [26]. NLRP upregulation results in cryopyrin-associated periodic syndromes (CAPS). NLRP3 gene mutations are typically autosomal dominant. In terms of severity, CAPS encompasses a range of diseases. Familial cold autoinflammatory syndrome (FCAS) is the least severe manifestation characterized by a cold-induced fever and urticaria-like rashes. The Muckle–Wells syndrome (MWS) is more severe and is associated with sensorineural hearing loss and arthritis. As the most common form of CAPS, chronic infantile neurological cutaneous arthritis (CINCA) has an infant onset and is associated with long bone epiphyseal overgrowth and chronic aseptic meningitis. Anti-IL 1 treatment is reported to have a remarkable effect on CAPS patients, particularly CINCA/NOMID patients and some RP patients [27,28].

NLRP12 pathogenesis was reported for the first time by Jeru in 2008 [29]. NLRP 12 modulates inflammation and tumor progression by inhibiting the production of proinflammatory cytokines and chemokines, as well as mitogen-activated protein kinase (MAPK) and extracellular signal-regulated kinase (ERK) signaling pathways. This protein is also important for the adaptative immune response as it regulates the migration of T lymphocytes, neutrophils, and dendritic cells, as well as the expression of molecules associated with the major histocompatibility complex (MHC) class I [13]. NLRP12 mutation can cause NLRP12-AID or familial cold-induced auto-inflammatory syndrome (FCAS2). However, the less-studied inflammasome NLRP12 appears to have a dual behavior—it is either anti-inflammatory by downregulating the production of numerous proinflammatory cytokines and chemokines, or proinflammatory, as is the case with certain pathogen infections (e.g., Yersinia), in which NLRP12 activates caspase-1, which results in a high amount of active IL-1ß and IL-18, similar to NLRP3 activation [23]. Although it may seem surprising, this duality is not new in fine-tuning the innate immune system, as IL-6 exhibits a similar characteristic [29,30,31,32,33]. As described by Normand et al., NLRP12 might also act as a potential inhibitor of NOD2 signaling in monocytes. Inflammatory conditions may result from monocytes lacking NLRP12 [34].

A recent literature review of NLRP12-related disease, which included cases from 2008 to 2022, has gathered 80 patients from around the world, including 33 pediatric cases. The most common symptom in children was periodic fever. This condition in the 33 children studied was accompanied by a variety of symptoms including joint pain, rash, abdominal pain, diarrhea, lymphadenopathy/splenomegaly, headache, neurosensory deafness, aphthous stomatitis, and acute-phase reactants elevations (in 55% of the cases) (Table 2). The age at diagnosis ranged from 2 months to 17-years-old and was predominant in females (20 cases, 65%). Up to 10 cases (30%) had symptoms induced by cold exposure. These patients had a number of comorbid diseases such as Crohn’s disease, C3 glomerulopathy, juvenile idiopathic arthritis, autoimmune hemolytic anemia, and susceptibility to infection [13].

In all cases, periodic fever was associated with abdominal discomfort (47% had pain and diarrhea), significant malnutrition (33%), and rarely, arthralgia and headache (20%). The possibility of recurrent infections may overlap with those of other primary immunodeficiencies or Crohn’s disease. As a result, when NLRP12 activity is associated with autoimmune diseases such as systemic lupus erythematous [35,36,37] and polyarthritis [38,39], this NOD-like receptor plays a critical role in the immune response to antigens.

The 33 pediatric cases of NLRP12-AID were caused by 21 different mutations—16 missense mutations (76%), 2 nonsense mutations (10%), and 3 frameshift mutations (14%). Mutated functional domains in NLRP12 diminish the predominant anti-inflammatory effect of NLRP through the unrestrained activation of NF-kB and enhanced caspase-1 activity. The most commonly reported mutation was c.1206C>G; pF402L. [13,29,40,41,42,43,44,45,46,47,48]. RP has not been described so far among the phenotypes associated with NLRP-AID, and nor has the association with the mutation presented in our patient. The NLRP12 variant identified in our patient (c.1264G>A) determines a replacement of glycine with arginine at codon 422 of the NLRP12 protein (p.Gly422Arg), a moderately conserved amino acid residue. This VUS variant has not been described in patients with NLRP12-AID, and nor is it present in population databases. It is difficult to predict a causative relationship between the identified variant and our patient’s phenotype consisting of fever, articular pain, pericardial and pleural effusion, increased CRP, ESR ferritin, and IL-1ß due to the lack of advanced functional studies. However, considering our findings, genetic studies of future RP cases might focus on NLRP12 activity as well.

Treatment aims to control hyperinflammation and improve the quality of life. This is usually not achieved by administering colchicine alone. Corticosteroid therapy transiently controls the disease. As a result of reducing the corticosteroid dose, the symptoms reappear. In its guidelines, the European Society of Cardiology (ESC) admits that the drugs used to treat pericardial disease are “off-label” [1]. There are four types of drugs that are effective in reducing the levels of IL-1ß. These include anakinra and canakinumab, which are approved in the European Union, as well as rilonacept and gevokizumab, which are not authorized in European countries [49].

Anakinra is a short-acting recombinant human IL-1R antagonist that can block the signaling pathway of IL-1 by competitively inhibiting the binding of IL-1ß and IL-1α to IL-1R. With FDA approval, anakinra can now be used to treat rheumatoid arthritis as well as CINCA-NOMID. In pediatric patients over the age of 8 months, anakinra has been approved by the European Union for treating juvenile rheumatoid arthritis, Still’s disease, and periodic fever syndrome. The number of pediatric NLRP12-AID patients treated with anakinra is relatively small. A case report of a 17-year-old female patient with NLRP12-AID who received anakinra was controlled rapidly and continuously after treatment initiation [41]. Another experimental study, conducted by Jeru [40], found a marked clinical improvement within two weeks of receiving anakinra treatment (1 mg/kg/d) in two brothers suffering from NLRP12-AID. In adults, the use of anakinra was studied in 224 patients with RP who were corticosteroid-dependent and colchicine-resistant. It was more than encouraging to see that the rate of recurrence was reduced by 83%. Most patients who were initially treated with anakinra discontinued any medication afterward. Upon follow-up of 18 months, 74% of these patients were free of recurrences, but 8.9% (20 patients) still required pericardiectomy. Following anakinra administration, corticosteroid therapy was continued at a low dosage in 61 patients (27%) [50]. Our patient responded very well to the anakinra treatment during the 48 months of follow-up, having only one relapse when, during the COVID-19 pandemic, the drug was not available, and the treatment was stopped but as soon as it was restarted no more relapses were registered.

Canakinumab is another specific drug. It has a long-acting effect as a humanized anti-monoclonal antibody. It binds to IL-1ß and prevents it from interacting with the IL-1 receptor. It is currently approved by the Food and Drug Administration for the treatment of periodic fever syndromes (including FMF, CAPS, HIDS, and MKD) in both adults and children over two years of age. Canakinumab was also approved for the treatment of Still’s disease and gouty arthritis by the European Union [51]. The drug’s half-life ranges from 21 to 28 days, and it can be injected once every eight weeks. There are few reports in the literature of the drug’s use—one case that was switched from anakinra to canakinumab due to local reactions associated with frequent subcutaneous injections with good response [42] and another case of an adult female for which the doses needed to be increased up to 5 mg/kg to obtain remission [48].

A new drug, Rilonacept, has been approved by the Food and Drug Administration (FDA). As an interleukin-1 inhibitor, rilonacept consists of two fusion proteins: the human interleukin-1 receptor component attached to the fragment-crystallizable portion, and the human interleukin-1 receptor component attached to the fragment-crystallizable portion and the human interleukin-1 receptor accessory protein linked to it [52]. Gevokizumab is an experimental monoclonal antibody that binds to IL-1ß and inhibits the signaling processes that cause cell inflammation. Its significance is not clearly defined in SAIDSs.

It was difficult to apply the treat-to-target concept to RP (and any other SAID), since there was no validated disease activity score. This problem was addressed by Ter Haar et al. and Gattorno et al. by developing and validating the autoinflammatory disease index and a diagnostic score, respectively [53,54,55].

For a long time, RP has been considered a localized inflammatory reaction. There are frequent reports of elevated levels of inflammatory markers (CRP, ESR, and more recently serum amyloid A) in these patients. As other authors have suggested, we should consider RP as a systemic disease, rather than a local one. A multidisciplinary approach should be adopted much earlier [13].

The absence of arthralgia and abdominal pain further underscores the unique and multifaceted presentation of recurrent pericarditis, shedding light on the atypical clinical features that can manifest in this condition.

This comprehensive evaluation reinforces the importance of considering a broad spectrum of clinical manifestations, whether positive or negative, when diagnosing and managing complex autoimmune and autoinflammatory diseases such as recurrent pericarditis.

The patient experienced a disease flare during the COVID-19 pandemic, raising questions about the potential relationship between viral infections and the course of recurrent pericarditis. It is important to note that the patient did not contract COVID-19 during this period. The disease flare occurred while the patient was still in therapy, and the specific timeline of discontinuation and relapse was as follows: The patient temporarily discontinued anakinra therapy for two weeks due to the unavailability of anakinra during the COVID-19 pandemic while the prednisone was already stopped due to the good response to anakinra. Subsequently, a disease flare was observed during this treatment hiatus.

Upon restarting anakinra therapy, the patient responded favorably, and no further relapses were registered. This observation underscores the importance of continued therapy in managing recurrent pericarditis and the potential impact of disruptions in treatment due to external factors, such as the COVID-19 pandemic. While the patient did not contract COVID-19, this case highlights the importance of maintaining consistent therapy for autoimmune and autoinflammatory conditions, particularly during external health crises, to ensure optimal disease management and control.

While an autosomal dominant mutation present in the father without clinical manifestations may initially seem surprising, several factors could contribute to this phenomenon. One possible explanation is the phenomenon of reduced penetrance, which is commonly observed in autosomal dominant disorders. Reduced penetrance refers to the situation where individuals with the same pathogenic mutation do not necessarily express the clinical features of the disease to the same extent or at all. Genetic modifiers, epigenetic factors, and environmental influences could play a role in determining the extent to which the mutation manifests clinically.

It is important to note that NLRP12-AID (NLRP12-associated autoinflammatory disease) can exhibit variable expressivity even within families. Therefore, while the father may carry the mutation, he may not have experienced clinical symptoms due to these complex genetic and environmental interactions.

Another possibility to consider is the age-related onset of symptoms. It is conceivable that the father may develop clinical manifestations at a later stage in life, as the age of onset for some autoinflammatory disorders can vary significantly.

Further research and genetic studies may shed light on the factors influencing the variable expressivity of NLRP12 mutations in different individuals within the same family. However, this case report emphasizes the importance of genetic screening and testing in family members, even in the absence of clinical symptoms, to identify potential carriers of mutations and facilitate an early intervention if needed.

## 4. Conclusions

Pediatric RP is a significant clinical issue due to the potential involvement of a dysregulated adaptive immune system and the difficulties in diagnosis and treatment. It is becoming increasingly evident that the innate immune system also plays a central role in generating and maintaining inflammation in the pericardium.

Clinical manifestations of autoinflammatory RP are similar to other etiologies, but the multiple recurrences of colchicine-resistant cortico-dependent pericarditis may offer a clue. More and more genetic studies are proving the role of the innate immune system by finding different mutations. We report a case of recurrent pericarditis associated with a missense NLRP12 mutation, which was previously not described in the medical literature, and had an excellent response to anakinra. The role of NLRP12 inflammasome in generating and maintaining recurrent pericardial inflammation should be considered.

## Figures and Tables

**Table 1:** Systemic autoinflammatory diseases.

Subgroup	Disease	Genetic Defect
Defects Affecting the Inflammasome	Muckle–Wells syndrome, Familial cold autoinflammatory syndrome 1 (FCAS1), Neonatal onset multisystem inflammatory disease (NOMID)	NLRP3
	Familial Mediterranean Fever (FMF)	MEFV
	Mevalonate kinase deficiency (Hyper IgD)	MVK
	NLRP1 deficiency or gain-of-function	NLRP1
	NLRC4-related disease (FCAS4)	NLRC4
	PLAID (PLCγ2 associated antibody deficiency and immune dysregulation)	PLCG2
	RIPK1 deficiency	RIPK1
	NLRP12-AID (FCAS2)	NLRP12
Non-Inflammasome Related Conditions	TNF receptor-associated periodic syndrome (TRAPS)	TNFRSF1A
	Pyogenic sterile arthritis, pyoderma gangrenosum, acne (PAPA) syndrome	PSTPP1
	Deficiency of the IL-1 receptor antagonist (DIRA)	IL1RN
	Deficiency of IL-36-receptor antagonist (DITRA)	IL36RN
	Majeed syndrome	LPIN-2
	Blau syndrome	NOD2
	CANDLE (chronic atypical neutrophilic dermatosis with lipodystrophy)	PSMG2
	Others	CARD14, COPA, ALP1, TRIM22
Type 1 Interferonopathies	ADA2 deficiency	ADA2
	STING-associated vasculopathy of infancy (SAVI) AR or AD forms	TMEM173
	Aicardi–Goutières syndrome 1–7	TREX1, RNASEH2, (A/B/C), SAMHD1, ADAR1, IFIH1, DNAse II, LSM 11, RNU7–1
	Very early onset pediatric lupus erythematosus	DNASE1L3
	Other	ACP5, POLA1, USP18, STAT2

**Table 2:** Clinical phenotype of NLRP12-AID in children.

Nr.	Symptoms and/or Other Manifestations	Percentage
1.	Periodic fever	100%
2.	Polyarthralgia/arthritis	55%
3.	Abdominal pain/diarrhea	48%
4.	Rash/urticaria	45%
5.	Lymphadenopathy/splenomegaly	33%
6.	Significant malnutrition	33%
7.	Headache	24%
8.	Neurosensory deafness	21%
9.	Aphthous stomatitis	12%
10.	Elevated acute-phase reactants	55%
11.	Infectious susceptibility	27%
12.	Autoimmune hemolytic anemia	3%
13.	C3 glomerulopathy	3%
14.	Interstitial lung disease	3%
15.	Crohn’s disease	9%
16.	Immunodeficiency	3%

## Data Availability

Not applicable.

## References

[1] Adler Y., Charron P., Imazio M., Badano L., Barón-Esquivias G., Bogaert J., Brucato A., Gueret P., Klingel K., Lionis C. (2015). 2015 ESC Guidelines for the diagnosis and management of pericardial diseases: The Task Force for the Diagnosis and Management of Pericardial Diseases of the European Society of Cardiology (ESC)Endorsed by: The European Association for Cardio-Thoracic Surgery (EACTS). Eur. Heart J..

[2] Brucato A., Imazio M., Cremer P.C., Adler Y., Maisch B., Lazaros G., Gattorno M., Caforio A.L.P., Marcolongo R., Emmi G. (2018). Recurrent pericarditis: Still idiopathic? The pros and cons of a well-honoured term. Intern. Emerg. Med..

[3] Andreis A., Imazio M., Casula M., Avondo S., Brucato A. (2021). Recurrent pericarditis: An update on diagnosis and management. Intern. Emerg. Med..

[4] Gouriet F., Levy P.Y., Casalta J.P., Zandotti C., Collart F., Lepidi H., Cautela J., Bonnet J.L., Thuny F., Habib G. (2015). Etiology of Pericarditis in a Prospective Cohort of 1162 Cases. Am. J. Med..

[5] Imazio M., Bobbio M., Cecchi E., Demarie D., Demichelis B., Pomari F., Moratti M., Gaschino G., Giammaria M., Ghisio A. (2005). Colchicine in addition to conventional therapy for acute pericarditis: Results of the COlchicine for acute PEricarditis (COPE) trial. Circulation.

[6] Imazio M., Brucato A., Cemin R., Ferrua S., Maggiolini S., Beqaraj F., Demarie D., Forno D., Ferro S., Maestroni S. (2013). A randomized trial of colchicine for acute pericarditis. N. Engl. J. Med..

[7] Imazio M., Bobbio M., Cecchi E., Demarie D., Pomari F., Moratti M., Ghisio A., Belli R., Trinchero R. (2005). Colchicine as first-choice therapy for recurrent pericarditis: Results of the CORE (COlchicine for REcurrent pericarditis) trial. Arch. Intern. Med..

[8] Imazio M., Brucato A., Cemin R., Ferrua S., Belli R., Maestroni S., Trinchero R., Spodick D.H., Adler Y. (2011). CORP (COlchicine for Recurrent Pericarditis) Investigators Colchicine for recurrent pericarditis (CORP): A randomized trial. Ann. Intern. Med..

[9] Imazio M., Belli R., Brucato A., Cemin R., Ferrua S., Beqaraj F., Demarie D., Ferro S., Forno D., Maestroni S. (2014). Efficacy and safety of colchicine for treatment of multiple recurrences of pericarditis (CORP-2): A multicentre, double-blind, placebo-controlled, randomised trial. Lancet.

[10] Brucato A., Brambilla G., Moreo A., Alberti A., Munforti C., Ghirardello A., Doria A., Shinar Y., Livneh A., Adler Y. (2006). Long-term outcomes in difficult-to-treat patients with recurrent pericarditis. Am. J. Cardiol..

[11] Caforio A.L., Brucato A., Doria A., Brambilla G., Angelini A., Ghirardello A., Bottaro S., Tona F., Betterle C., Daliento L. (2010). Anti-heart and anti-intercalated disk autoantibodies: Evidence for autoimmunity in idiopathic recurrent acute pericarditis. Heart.

[12] Tangye S.G., Al-Herz W., Bousfiha A., Cunningham-Rundles C., Franco J.L., Holland S.M., Klein C., Morio T., Oksenhendler E., Picard C. (2022). Human Inborn Errors of Immunity: 2022 Update on the Classification from the International Union of Immunological Societies Expert Committee. J. Clin. Immunol..

[13] Wang H.F. (2022). NLRP12-associated systemic autoinflammatory diseases in children. Pediatr. Rheumatol. Online J..

[14] Soler-Soler J., Sagristà-Sauleda J., Permanyer-Miralda G. (2004). Relapsing pericarditis. Heart.

[15] Chiabrando J.G., Bonaventura A., Vecchié A., Wohlford G.F., Mauro A.G., Jordan J.H., Grizzard J.D., Montecucco F., Berrocal D.H., Brucato A. (2020). Management of Acute and Recurrent Pericarditis: JACC State-of-the-Art Review. J. Am. Coll. Cardiol..

[16] Kelley N., Jeltema D., Duan Y., He Y. (2019). The NLRP3 Inflammasome: An Overview of Mechanisms of Activation and Regulation. Int. J. Mol. Sci..

[17] Pathak S., McDermott M.F., Savic S. (2017). Autoinflammatory diseases: Update on classification diagnosis and management. J. Clin. Pathol..

[18] Havnaer A., Han G. (2019). Autoinflammatory Disorders: A Review and Update on Pathogenesis and Treatment. Am. J. Clin. Dermatol..

[19] Takeuchi O., Akira S. (2010). Pattern recognition receptors and inflammation. Cell.

[20] Sharma D., Kanneganti T.D. (2016). The cell biology of inflammasomes: Mechanisms of inflammasome activation and regulation. J. Cell Biol..

[21] Lamkanfi M., Dixit V.M. (2014). Mechanisms and functions of inflammasomes. Cell.

[22] Minkiewicz J., de Rivero Vaccari J.P., Keane R.W. (2013). Human astrocytes express a novel NLRP2 inflammasome. Glia.

[23] Vladimer G.I., Weng D., Paquette S.W., Vanaja S.K., Rathinam V.A., Aune M.H., Conlon J.E., Burbage J.J., Proulx M.K., Liu Q. (2012). The NLRP12 inflammasome recognizes Yersinia pestis. Immunity.

[24] Cochino A.V., Ioan A., Farkas O.M. (2022). Autoinflammatory Diseases in Romanian Children: A Tertiary Center Case Series Study. J. Clin. Rheumatol. Pract. Rep. Rheum. Musculoskelet. Dis..

[25] Lopalco G., Rigante D., Cantarini L., Imazio M., Lopalco A., Emmi G., Venerito V., Fornaro M., Frediani B., Nivuori M. (2021). The autoinflammatory side of recurrent pericarditis: Enlightening the pathogenesis for a more rational treatment. Trends Cardiovasc. Med..

[26] Mauro A.G., Bonaventura A., Vecchié A., Mezzaroma E., Carbone S., Narayan P., Potere N., Cannatà A., Paolini J.F., Bussani R. (2021). The Role of NLRP3 Inflammasome in Pericarditis: Potential for Therapeutic Approaches. JACC. Basic Transl. Sci..

[27] Peet C.J., Rowczenio D., Omoyinmi E., Papadopoulou C., Mapalo B.R.R., Wood M.R., Capon F., Lachmann H.J. (2022). Pericarditis and Autoinflammation: A Clinical and Genetic Analysis of Patients With Idiopathic Recurrent Pericarditis and Monogenic Autoinflammatory Diseases at a National Referral Center. J. Am. Heart Assoc..

[28] Karacan İ., Balamir A., Uğurlu S., Aydın A.K., Everest E., Zor S., Önen M.Ö., Daşdemir S., Özkaya O., Sözeri B. (2019). Diagnostic utility of a targeted next-generation sequencing gene panel in the clinical suspicion of systemic autoinflammatory diseases: A multi-center study. Rheumatol. Int..

[29] Jéru I., Duquesnoy P., Fernandes-Alnemri T., Cochet E., Yu J.W., Lackmy-Port-Lis M., Grimprel E., Landman-Parker J., Hentgen V., Marlin S. (2008). Mutations in NALP12 cause hereditary periodic fever syndromes. Proc. Natl. Acad. Sci. USA.

[30] Alonzi T., Fattori E., Lazzaro D., Costa P., Probert L., Kollias G., De Benedetti F., Poli V., Ciliberto G. (1998). Interleukin 6 is required for the development of collagen-induced arthritis. J. Exp. Med..

[31] Yamamoto M., Yoshizaki K., Kishimoto T., Ito H. (2000). IL-6 is required for the development of Th1 cell-mediated murine colitis. J. Immunol..

[32] Xing Z., Gauldie J., Cox G., Baumann H., Jordana M., Lei X.F., Achong M.K. (1998). IL-6 is an antiinflammatory cytokine required for controlling local or systemic acute inflammatory responses. J. Clin. Investig..

[33] Borghini S., Tassi S., Chiesa S., Caroli F., Carta S., Caorsi R., Fiore M., Delfino L., Lasigliè D., Ferraris C. (2011). Clinical presentation and pathogenesis of cold-induced autoinflammatory disease in a family with recurrence of an NLRP12 mutation. Arthritis Rheum..

[34] Normand S., Waldschmitt N., Neerincx A., Martinez-Torres R.J., Chauvin C., Couturier-Maillard A., Boulard O., Cobret L., Awad F., Huot L. (2018). Proteasomal degradation of NOD2 by NLRP12 in monocytes promotes bacterial tolerance and colonization by enteropathogens. Nat. Commun..

[35] Hoffman H.M., Mueller J.L., Broide D.H., Wanderer A.A., Kolodner R.D. (2001). Mutation of a new gene encoding a putative pyrin-like protein causes familial cold autoinflammatory syndrome and Muckle-Wells syndrome. Nat. Genet..

[36] Simon A., van der Meer J.W., Vesely R., Myrdal U., Yoshimura K., Duys P., Drenth J.P. (2006). International HIDS Study Group Approach to genetic analysis in the diagnosis of hereditary autoinflammatory syndromes. Rheumatology.

[37] Chen M., Tsao Y., Chen S. (2019). NLRP12 Regulates Interferon-α Expression and Is a Biomarker for Disease Activity of Systemic Lupus Erythematosus [abstract]. Arthritis Rheumatol..

[38] Vance E.E., Lee E.L., Brown B., Snow P.E., Truax A., Ting J.P., Caspi R.R., Rosenzweig H.L. (2016). The innate immune receptor Nlrp12 participates in protection against experimental autoimmune uveitis. Investig. Ophthalmol. Vis. Sci..

[39] Ayla A.Y., Eren H., Zare J., Calhan S.S., Karacan I., Seven M., Ugurlu S. (2021). A rare case of an NLRP12-associated autoinflammatory disease. Eur. J. Med. Genet..

[40] Jéru I., Le Borgne G., Cochet E., Hayrapetyan H., Duquesnoy P., Grateau G., Morali A., Sarkisian T., Amselem S. (2011). Identification and functional consequences of a recurrent NLRP12 missense mutation in periodic fever syndromes. Arthritis Rheum..

[41] Vitale A., Rigante D., Maggio M.C., Emmi G., Romano M., Silvestri E., Lucherini O.M., Emmi L., Gerloni V., Cantarini L. (2013). Rare NLRP12 variants associated with the NLRP12-autoinflammatory disorder phenotype: An Italian case series. Clin. Exp. Rheumatol..

[42] Başaran Ö., Uncu N., Çakar N., Turanlı E.T., Kiremitci S., Aydın F., Bayrakcı U.S. (2018). C3 glomerulopathy in NLRP12-related autoinflammatory disorder: Case-based review. Rheumatol. Int..

[43] Ghosh K., Mishra K., Shah A., Patel P., Shetty S. (2019). Novel Deleterious Sequence Change in the NLRP12 Gene in a Child with the Autoinflammatory Syndrome, Joint Hypermobility and Cutis Laxa from India. Mediterr. J. Hematol. Infect. Dis..

[44] Gupta L., Ahmed S., Singh B., Prakash S., Phadke S., Aggarwal A. (2021). Novel NLRP12 variant presenting with familial cold autoimmunity syndrome phenotype. Ann. Rheum. Dis..

[45] Wang W., Zhou Y., Zhong L.Q., Li Z., Jian S., Tang X.Y., Song H.M. (2020). The clinical phenotype and genotype of NLRP12-autoinflammatory disease: A Chinese case series with literature review. World J. Pediatr. WJP.

[46] Wang W., Yu Z., Gou L., Zhong L., Li J., Ma M., Wang C., Zhou Y., Ru Y., Sun Z. (2020). Single-Center Overview of Pediatric Monogenic Autoinflammatory Diseases in the Past Decade: A Summary and Beyond. Front. Immunol..

[47] Yang X., Zhao B., Liao Y. (2023). Analysis on misdiagnosis of a case of novel variant of NLRP12. Ann. Rheum. Dis..

[48] Kostik M.M., Suspitsin E.N., Guseva M.N., Levina A.S., Kazantseva A.Y., Sokolenko A.P., Imyanitov E.N. (2018). Multigene sequencing reveals heterogeneity of NLRP12-related autoinflammatory disorders. Rheumatol. Int..

[49] Bettiol A., Lopalco G., Emmi G., Cantarini L., Urban M.L., Vitale A., Denora N., Lopalco A., Cutrignelli A., Lopedota A. (2019). Unveiling the efficacy, safety, and tolerability of anti -Interleukin -1 treatment in monogenic and multifactorial autoinflammatory diseases. Int. J. Mol. Sci..

[50] Imazio M., Andreis A., De Ferrari G.M., Cremer P.C., Mardigyan V., Maestroni S., Luis S.A., Lopalco G., Emmi G., Lotan D. (2020). Anakinra for corticosteroid-dependent and colchicine-resistant pericarditis: The IRAP (International Registry of Anakinra for Pericarditis) study. Eur. J. Prev. Cardiol..

[51] Hausmann J.S. (2019). Targeting cytokines to treat autoinflammatory diseases. Clin Immunol..

[52] European Medicine Agency (EMA) (2022). Rilonacept Regeneron.

[53] Ter Haar N.M., Annink K.V., Al-Mayouf S.M., Amaryan G., Anton J., Barron K.S., Benseler S.M., Brogan P.A., Cantarini L., Cattalini M. (2017). Development of the autoinflammatory disease damage index (ADDI). Ann. Rheum. Dis..

[54] Ter Haar N.M., van Delft A.L.J., Annink K.V., van Stel H., Al-Mayouf S.M., Amaryan G., Anton J., Barron K.S., Benseler S., Brogan P.A. (2018). In silico validation of the Autoinflammatory Disease Damage Index. Ann. Rheum. Dis..

[55] Gattorno M., Sormani M.P., D’Osualdo A., Pelagatti M.A., Caroli F., Federici S., Cecconi M., Solari N., Meini A., Zulian F. (2008). A diagnostic score for molecular analysis of hereditary autoinflammatory syndromes with periodic fever in children. Arthritis Rheum..

